# Risk Stratification for Bipolar Disorder Using Polygenic Risk Scores Among Young High-Risk Adults

**DOI:** 10.3389/fpsyt.2020.552532

**Published:** 2020-10-26

**Authors:** Silvia Biere, Thorsten M. Kranz, Silke Matura, Kristiyana Petrova, Fabian Streit, Andreas G. Chiocchetti, Oliver Grimm, Murielle Brum, Natalie Brunkhorst-Kanaan, Viola Oertel, Aliaksandr Malyshau, Andrea Pfennig, Michael Bauer, Thomas G. Schulze, Sarah Kittel-Schneider, Andreas Reif

**Affiliations:** ^1^Department of Psychiatry, Psychosomatic Medicine and Psychotherapy, University Hospital, Goethe University, Frankfurt, Germany; ^2^Department of Genetic Epidemiology in Psychiatry, Central Institute of Mental Health, Medical Faculty Mannheim/University of Heidelberg, Mannheim, Germany; ^3^Department of Child and Adolescent Psychiatry, Psychosomatics and Psychotherapy, Autism Research and Intervention Center of Excellence Frankfurt, University Hospital Frankfurt, Goethe University, Frankfurt am Main, Germany; ^4^Department of Psychiatry and Psychotherapy, Carl Gustav Carus University Hospital, Dresden University of Technology, Dresden, Germany; ^5^Institute of Psychiatric Phenomics and Genomics, University Hospital Munich, Ludwig-Maximilians-University of Munich, Munich, Germany; ^6^Department of Psychiatry, Psychosomatic Medicine and Psychotherapy, University Hospital of Würzburg, University of Würzburg, Würzburg, Germany

**Keywords:** polygenic risk score, bipolar disorder, genetic phenotypes, depression, ADHD, early recognition

## Abstract

**Objective:** Identifying high-risk groups with an increased genetic liability for bipolar disorder (BD) will provide insights into the etiology of BD and contribute to early detection of BD. We used the BD polygenic risk score (PRS) derived from BD genome-wide association studies (GWAS) to explore how such genetic risk manifests in young, high-risk adults. We postulated that BD-PRS would be associated with risk factors for BD.

**Methods:** A final sample of 185 young, high-risk German adults (aged 18–35 years) were grouped into three risk groups and compared to a healthy control group (*n* = 1,100). The risk groups comprised 117 cases with attention deficit hyperactivity disorder (ADHD), 45 with major depressive disorder (MDD), and 23 help-seeking adults with early recognition symptoms [ER: positive family history for BD, (sub)threshold affective symptomatology and/or mood swings, sleeping disorder]. BD-PRS was computed for each participant. Logistic regression models (controlling for sex, age, and the first five ancestry principal components) were used to assess associations of BD-PRS and the high-risk phenotypes.

**Results:** We observed an association between BD-PRS and combined risk group status (OR = 1.48, *p* < 0.001), ADHD diagnosis (OR = 1.32, *p* = 0.009), MDD diagnosis (OR = 1.96, *p* < 0.001), and ER group status (OR = 1.7, *p* = 0.025; not significant after correction for multiple testing) compared to healthy controls.

**Conclusions:** In the present study, increased genetic risk for BD was a significant predictor for MDD and ADHD status, but not for ER. These findings support an underlying shared risk for both MDD and BD as well as ADHD and BD. Improving our understanding of the underlying genetic architecture of these phenotypes may aid in early identification and risk stratification.

## Introduction

Bipolar disorder (BD), which is characterized by recurrent episodes of mania and depression, is a severe and often chronic mental disorder associated with increased premature mortality and disability and reduced quality of life (1, 2). The first symptoms of the disorder occur many years before patients meet full diagnostic criteria, typically in adolescence, which thus marks a high-risk period for BD onset (3, 4). The mean age of onset for BD is between 20 and 30 years, and risk of onset decreases with age thereafter (1, 2). The long interval between early symptoms, correct diagnosis, and adequate treatment (5.8–6.7 years) (5, 6) is associated with a worsened clinical course and a substantial burden of illness (7, 8).

In the early course of BD, mood and drive are often dysregulated (9), which manifests in episodes of (subclinical) depression as well as (sub)threshold hypomania—these increase in severity and frequency during the period until onset (4, 6, 10). While the abovementioned symptoms are difficult to differentiate from normal fluctuations in mood (5), they represent the best predictors for developing BD (4, 10, 11). Additional symptoms include sleep disturbances, fear, anger, and irritability, which often occur in the early course and become more specific and similar to BD symptoms over time (4, 8, 9, 12).

Other difficulties that contribute to misdiagnosis of BD include a high rate of comorbidity and substantial overlap of symptoms between BD and other psychiatric disorders (13). The lifetime prevalence of attention deficit hyperactivity disorder (ADHD) in bipolar patients has been estimated to be around 20% and is thus one of the most common comorbid disorders in BD (14–16). ADHD has an earlier age of onset than BD and is common in relatives and offspring of individuals with BD, which has led to the hypothesis that it may be a precursor of BD (17, 18). However, while there are inconsistent findings regarding a genetic overlap between ADHD and BD (19), recent studies assessing genetic correlations between large-scale genome-wide association studies (GWAS) indicate a modest but significant positive association (12, 18, 20).

In most BD patients, the first episode at the onset of the disorder is a depressive episode, whereas an index mood episode of (hypo-)mania is less likely (21–23). Early age at onset of the first depressive disorder seems to be a prominent risk factor of conversion to BD (24–26). The difficulty in distinguishing BD from major depressive disorder (MDD) before the first (hypo-)manic episode occurs implies that BD diagnosis is often preceded by an initial misdiagnosis of MDD (21). This phenomenon creates the category of the so-called “hidden bipolars.” Observed conversion rates from MDD to BD in young adults varies between 2.5 and 15.4% in a follow-up interval of 3–9 years (24, 27, 28). Moreover, studies have provided considerable support for a high shared genetic risk between BD and MDD (29). Family history for BD has been found to be the strongest predictor for conversion (30).

With heritability rates of up to 70% for BD (31), understanding the genetic factors contributing to BD-specific symptoms is crucial to improving diagnosis. Early and accurate diagnosis of BD would aid timely intervention and potentially prevent serious consequential damage. GWAS focusing on the liability for BD have identified shared risk alleles of single-nucleotide polymorphisms (SNPs) between BD and MDD as well as between BD and ADHD (32–35). While individual SNPs have a very small effect on disease risk on their own, a polygenic risk score (PRS), which constitutes a single value estimate of an individual's genetic propensity to a phenotype across a vast array of SNPs, appears to be a promising improvement (36). The PRS is the sum of an individual's genome-wide additive risk for a certain phenotype based on variation in multiple genetic loci and their associated weights from GWAS. Thus, for complex genetic diseases such as BD, PRS are likely to become a valuable predictor for disease risk. PRS using information from disease-associated alleles on current GWAS platforms explain ~4% of the variation in risk for BD on the liability scale (29, 31). The modest accuracy of PRS is likely due to the highly polygenic nature of psychiatric disorders (37). In the future, higher levels of prediction accuracy may be achieved with predictors estimated from very large discovery samples (38). As GWAS datasets become larger and more diverse, they will have valuable potential for genomic risk prediction (39). Currently, PRS analyses are one of the most widely used approaches to understanding the genetic overlap between disorders, as well as at symptom level in case–control target samples (37).

To improve prognosis or even prevent the development of full-blown BD for affected individuals, there is a clear need to identify causative factors in order to improve diagnosis in the early stages of BD (2, 6). The present study investigates whether BD-PRS is associated with specific prodromal risk groups for BD. Based on previous research, we recruited subjects aged 18–35 belonging to three phenotypic risk groups for BD: Subjects with either ADHD or MDD diagnosis or early recognition (ER) risk factors assessed with the Early Phase Inventory for Bipolar Disorders (EPIbipolar) (13). These groups are being followed up longitudinally to assess the interplay of genetic and clinical predictors for pre-diagnostic risk stratification for BD (40).

## Materials and Methods

### Participants

The study sample comprised 203 high-risk young adults aged 18–35 either diagnosed with ADHD (*n* = 128) or MDD (*n* = 51) or belonging to the ER group (*n* = 24) using standardized instruments (see *Clinical assessments*). Of these, 112 (ADHD = 32, MDD = 56, ER = 24) were recruited as part of the BipoLife substudy “Improving early recognition and intervention in people at-risk of developing bipolar disorder (BD),” which monitors young help-seeking adults over a 3-years period (40, 41). The additional young ADHD adults (*n* = 96) originated from the “Comorbid Conditions of Attention deficit hyperactivity disorders (CoCA)” study, which focuses on the investigation of developmental pathways, genetic and environmental mechanisms that underlie comorbidity of ADHD (42). The control group consisted of 1,223 healthy subjects with no history of psychiatric disorders from the longitudinal resilience assessment (LORA) project (https://lora-studie.de/) investigating the mechanisms involved in the resilience process as they occur in response to the stressors of modern life over a 3-years period (43).

All subjects declared that they understood the experimental procedure and provided written informed consent. The study was undertaken in accordance with the Code of Ethics of the World Medical Association (Declaration of Helsinki; Rickham, 2013) and approved by the Ethics Committee of the University Hospital Frankfurt am Main, Germany. All subjects were recruited at the Department of Psychiatry, Psychosomatic Medicine and Psychotherapy at the University Hospital Frankfurt.

### Clinical Assessments

The inclusion criteria for high-risk subjects were a DSM-IV or DSM-5 diagnosis of either MDD or ADHD or classification into an ER risk group and age in the range of 18–35 years. After an initial screening visit of all participants, the German version of the Structured Clinical Interview for DSM-IV Axis I disorders (SCID-I) (44) was carried out with all potential high-risk subjects. Individuals who fulfilled the criteria for a diagnosis of BD, schizoaffective disorder, or schizophrenia as well as those suffering exclusively from substance abuse, anxiety disorder, or obsessive–compulsive disorder were excluded. A comorbid personality disorder was not an exclusion criterion. All remaining subjects were assigned to one of the three risk groups (ADHD, MDD, and ER) depending on diagnosis.

To be assigned to the ADHD risk group, participants needed to fulfill the DSM-5 criteria for ADHD assessed by the DIVA questionnaire (45) and score above the cutoffs in the ADHD self-rating scales (German “ADHS-SB”) (46). If available, external evaluation from family members/colleagues for ADHD was also considered. In addition, scores of 30 or above on the short version of the German version of the Wender-Utah-Rating Scale (WURS-k) (47) for retrospective childhood symptoms were required. To be assigned to the MDD risk group, subjects needed to fulfill the criteria for a MDD diagnosis in the SCID-I. For young, help-seeking adults that did not have a confirmed SCID-I diagnosis, the risk assessment tool EPIbipolar was used to assign participants to the ER risk group (13). EPIbipolar operationalizes risk constellations out of the elevated risk factors that are associated with later conversion to BD [(I) positive family history for BD, (II) (sub)threshold affective symptoms, (III) mood swings, (IV) changes in sleep and circadian rhythm, (V) substance misuse or dependence, (VI) impairment in psychosocial functioning, (VII) fearfulness/anxiety, and (VIII) episodic course] and forms risk groups. We assumed that elevated risk might be captured best by including all participants meeting the criteria for the risk categories defined in EPIbipolar (risk group, high-risk group, and ultra-high-risk group) and exclude subjects with no risk group assignment only. Only one participant from the high-risk group, who did not meet the criteria for ADHD or MDD or any of the risk categories defined in EPIbipolar was excluded from the final regression analyses.

Current or past psychiatric symptoms were ascertained in healthy controls to rule out an axis-I disorder (according to DSM-IV and DSM-5, respectively) by semi-structured interview with the Mini-International Neuropsychiatric Interview (M.I.N.I.) (48). All diagnostic interviews were conducted by trained and experienced clinicians.

### Genotyping and Quality Control

Genotyping was performed using the Global Screening Array (GSA), Multiple Drops (MD) Version 2.0 at the Life & Brain GmbH Platform Genomics, Bonn, Germany for the 51 MDD and 24 ER subjects. Genotyping of the 128 ADHD cases and 1,223 controls was carried out on a GSA-MD V 1.0 at the Broad Institute in Cambridge, Massachusetts, USA. Quality control of all subjects was performed using PLINK v1.9 (49). SNPs were filtered to exclude those with minor allele frequencies ≤ 0.01, calling rate of ≤ 0.98, variants deviating from Hardy–Weinberg-Equilibrium (HWE) (*p* < 1 × 10^−6^), and tri-allelic variants or variants not uniquely mappable. Participants were excluded in case of missingness >0.02, heterozygosity rate > 0.2, and sex mismatch. Filtering for population structure and relatedness was carried out on selected high-quality (HWE *p* < 0.02, MAF >0.2, missingness = 0) SNP set that was LD pruned (*r*^2^ = 0.1). In case of cryptically related subjects (pi hat > 0.2), one of the subjects was excluded, preferentially retaining cases. Principal component analysis (PCA) was performed to assess hidden population stratification, and outliers with a *SD* > 6 on one of the first 20 principal components were excluded. After quality control, the datasets were merged and another round of quality control and PCA were carried out as described above.

In total, 141 subjects were excluded from subsequent analyses: 117 subjects were excluded after genetic quality control, 22 subjects were excluded because of missing information on age, and 1 ER subject was excluded for not fulfilling the criteria for ER risk group status (see *Clinical assessments*). The final dataset thus consisted of *N* = 1,285 participants (117 ADHD, 45 MDD, and 23 ER high-risk subjects and 1,100 healthy controls) and 431,828 SNPs.

### Polygenic Risk Scores

PRS calculated was performed using the PRSice software version 2.3.1.e with default options [clump-kb 250, clump-p 1.0, clump r2 0.1, interval 5e-05, lower 5e-08, stat OR; (50)]. We calculated PRS for BD based on the summary statistic files of the second Psychiatric Genomics Consortium Bipolar Disorder (PGC-BD) GWAS (31), applying INFO score filtering (INFO > 0.8). There was no overlap between the present study sample and the used BD discovery sample. BD-PRS were z-transformed based on the mean and standard deviation observed in the control group. We applied the best-fit function of PRSice, which runs logistic regressions to determine the p-threshold with the largest variance explained by the PRS, assessed as the increment in Nagelkerke's pseudo-*R*^2^ of the full model including BD-PRS and covariates (age, sex, and the first five principal components for population stratification) compared to the null model (only covariates). The best-fit PRS for the combined sample (dependent variable: high-risk vs. control group) was used for further subgroup comparisons. In addition to the incremental *R*^2^ values, we report the incremental *R*^2^ adjusted for the liability scale (onwards referred to as “R2.liability”; –prevalence flag in PRSice2), assuming a more conservative estimated population lifetime prevalence of 2.5% for ADHD in adults (51) and 15% for MDD (52), as well as 17% for ER (unpublished data). For the combined risk group, we applied an average prevalence weighted by the subsample sizes as an approximation of prevalence (7.39%).

### Statistics

All further analyses were performed using SPSS 26.0 for Windows (IBM Corp., USA). To examine if BD-PRS (the independent variable) was associated with a specific risk group compared to control status (dependent variable), binary logistic regressions were carried out. Odds ratios (ORs) per standard deviation (SD) increase in BD-PRS are reported. Each regression included sex, age, and the first five principal components (to control for hidden population stratification) as covariates. Uncorrected *p*-values are reported, thus the corresponding Bonferroni-corrected alpha threshold was 0.0125 (correcting for four analyses, i.e. any risk group vs. control, ADHD vs. control, MDD vs. control and ER vs. control).

Given that the sample size was pre-defined at the beginning of the study, we performed a *post-hoc* power analysis to identify the beta error with the given sample size. For the four different binary logistic regressions, we calculated the *post-hoc* statistical power for the estimated population effect sizes with GPower 3.1.9.7 (53) for an population effect of an *R*^2^ of 0.04 (corresponding to a Cohen's d of 0.41) as observed in the PRS analyses in the GWAS by Stahl et al. (31) using the following parameters: Corrected alpha error probability of 0.0125, control group sample size of *n* = 1,100, and experimental group sample size of *n* = 185 (combined risk group), *n* = 117 (ADHD), *n* = 45 (MDD), and *n* = 23 (ER), respectively. Power analysis revealed a statistical power of >0.99 for the regression with the combined risk group, and 0.95 (ADHD group), 0.57 (MDD group), and 0.30 (ER group).

## Results

### Sample Characteristics

The age of the participants (39.4% male, 60.6% female) at the time of the interview ranged from 18 to 82 years, with a mean of 31.39 (*SD* = 12.65) years (Table 1).

**Table 1:** Demographic data.

**Demographics**	**ADHD (*N* = 117)**	**MDD (*N* = 45)**	**ER (*N* = 23)**	**Control (*N* = 1,100)**
**Sex (%)**				
Female	41%	64.4%	65.2%	62.6%
Male	59%	35.6%	34.8%	37.4%
Age (years ± SD)	27.21 ± 4.60	25.07 ± 4.83	23.39 ± 4.79	32.30 ± 13.59

*Control, healthy control; ADHD, attention deficit hyperactivity disorder; MDD, major depressive disorder; ER, early recognition; SD, standard deviation*.

### Risk Group Association With Genetic Risk of BD

**Figure 1:**
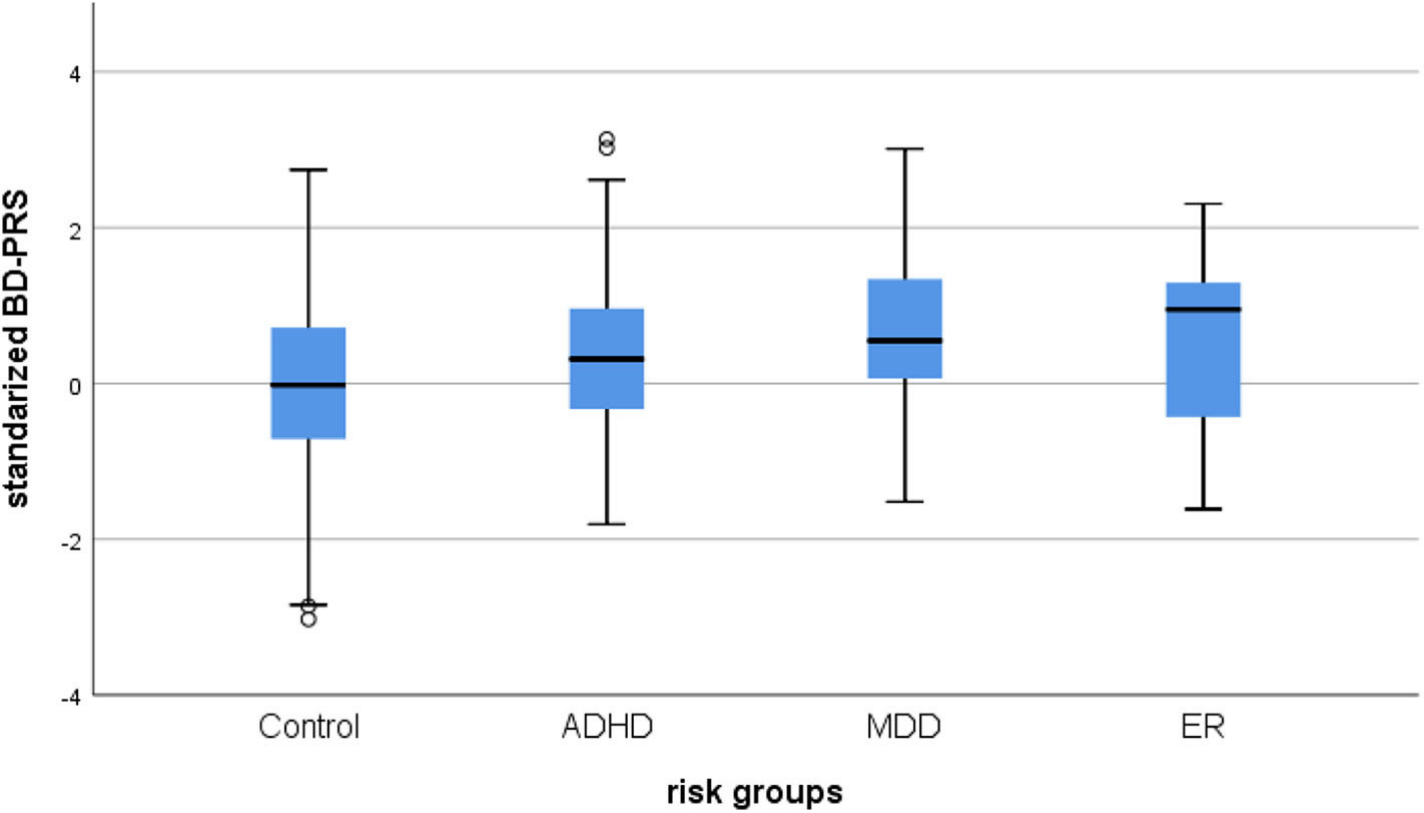
Box plot of the distribution of BD-PRS for BD high-risk groups. Control, healthy control group; ADHD, attention deficit hyperactivity disorder; MDD, major depressive disorder; ER, early recognition; ZScore: BD-PRS, standardized BD-PRS score.

BD-PRS score was positively associated with belonging to the combined risk group (OR = 1.48, 95% CI [1.25, 1.76], PRS.*R*^2^ =0.026, PRS.*R*^2^.liability =0.030, *p* < 0.001) vs. controls. Binary logistic regression for the specific subgroups showed BD-PRS was also associated with ADHD vs. control status (OR = 1.32, 95% CI [1.07, 1.62], PRS.*R*^2^ = 0.011, PRS. *R*^2^.liability = 0.011, *p* = 0.009) and MDD vs. control status (OR = 1.96, 95% CI [1.42, 2.73], PRS.*R*^2^ = 0.050, PRS.*R*^2^.liability = 0.094, *p* < 0.001). A trend was observed for BD-PRS prediction of ER group vs. control status (OR = 1.70, 95% CI [1.07, 2.69], PRS.*R*^2^ = 0.024, PRS *R*^2^.liability = 0.049; *p* = 0.025, see Figure 1). Only the association with ER vs. control group status was not significant per the Bonferroni-corrected alpha level of 0.0125. None of the analyzed samples showed hidden population stratification in the first five principal components (PC1–PC5). For a summary of the regression coefficients, see Table 2.

**Table 2:** Associations of the BD-PRS with risk groups.

**Risk group**	**β**	***SE***	***P***	***OR***	**95% CI**	**Nagelkerke's *R*^**2**^ observed**	**Nagelkerke's *R*^**2**^ liability**
**All risk groups (*****N*** **=** **185)**						0.026	0.030
BD-PRS	0.39	0.087	<0.001^*^	1.48	1.25–1.76		
Sex	−0.58	0.17	0.001^*^	0.56	0.41–0.78		
Age	0.066	0.012	<0.001^*^	0.94	0.92–0.96		
PC 1	−2.596	2.56	0.310	0.075	0.000–11.2		
PC 2	−1.35	2.67	0.615	0.26	0.001–48.9		
PC 3	−0.75	2.86	0.794	0.47	0.002–129		
PC 4	−3.10	2.98	0.298	0.045	0.000–15.5		
PC 5	5.53	2.96	0.062	252	0.76–83,406		
**ADHD (*****n*** **=** **117)**							
BD-PRS	0.28	0.11	0.009^*^	1.32	1.07–1.62	0.011	0.011
Sex	−0.92	0.20	<0.001^*^	0.40	0.27–0.59		
Age	−0.048	0.012	<0.001^*^	0.95	0.93–0.98		
PC 1	−4.23	2.92	0.148	0.02	0.000–4.50		
PC 2	−1269	3.12	0.684	0.28	0.001–128		
PC 3	−0.13	3.44	0.970	0.88	0.001–747		
PC 4	−2.62	3.66	0.474	0.073	0.000–95.0		
PC 5	0.22	3.54	0.951	1.245	0.001–1277		
**MDD (*****n*** **=** **45)**							
BD-PRS	0.68	0.17	<0.001^*^	1.96	1.42–2.73	0.050	0.094
Sex	0.099	0.31	0.765	1.10	0.58–2.11		
Age	−0.090	0.027	0.001^*^	0.91	0.87–0.96		
PC 1	2.51	5.02	0.616	12.4	0.001–229298		
PC 2	−4.38	4.76	0.358	0.01	0.000–141		
PC 3	−2.05	5.14	0.689	0.13	0.000–3018		
PC 4	−4.66	5.76	0.419	0.01	0.000–760		
PC 5	15.3	5.72	0.007	4389555	59.7–3.23E+11		
**ER (*****n*** **=** **23)**							
BD-PRS	0.53	0.24	0.025	1.70	1.07–2.69	0.02	0.05
Sex	0.080	0.46	0.862	1.08	0.44–2.66		
Age	−0.17	0.054	0.002^*^	0.85	0.76–0.94		
PC 1	0.64	7.13	0.928	1.90	0.000–2238258		
PC 2	9.42	8.28	0.255	12377	0.001– 1.38E+11		
PC 3	0.74	8.08	0.927	2.10	0.000–15892441		
PC 4	2.79	7.72	0.718	16	0.000–60807382		
PC 5	15.9	7.86	0.043	8055215	1.64–3.95E+13		

*Binary logistic regressions were adjusted for sex, age, and ancestry PCs 1–5. Sex was coded as 1 = male and 2 = female*.

* *BD-PRS significant after applying the corrected alpha threshold of 0.0125*.

*ADHD, attention deficit hyperactivity disorder; MDD, major depressive disorder; ER, early recognition; CI, confidence interval; PC, principal component*.

## Discussion

### Summary of Findings

To date, few studies have investigated phenotypes associated with genetic risk of BD utilizing BD-PRS (19, 54). In this study, we uniquely investigated the role of psychopathology and high-risk factors in young adults for the development of BD using BD-PRS scores. Our results provide information about shared genetic risk factors, supporting the hypothesis that BD-PRS might improve the accuracy of BD diagnosis in the early course of illness or prodromal phase. Overall, the results for the combined risk group (ADHD, MDD, and ER) displayed a weak association between the BD-PRS and the respective diagnoses, as made evident by the expected risk-increasing profile (OR = 1.48). In the subgroup analyses, BD-PRS was a significant predictor of both MDD and ADHD diagnosis vs. healthy control status in young adults, but not of ER group status. For MDD as well as ADHD, we observed a weak risk association of BD-PRS and case vs. healthy control status (OR_MDD_ = 1.96; OR_ADHD_ = 1.32). BD-PRS did not show a significant association with ER group status as per the Bonferroni-corrected significance level, which may be due to the limited statistical power for this comparatively small subsample. The results from our high-risk young adult cohort indicate that the expected shared risk between both MDD and BD and ADHD and BD is considerable. Although the discovery GWAS sample used to calculate BD-PRS in our study is the largest available to date, the predictive power achievable by polygenic scores for BD is still limited. Future, large-scale GWAS will enable better prediction of polygenic risk for developing BD and aid accurate diagnosis.

### BD-PRS and MDD

The results of the present study are consistent with and extend previous findings of a strong genetic overlap between BD and MDD (54, 55). From a clinical perspective, one possible explanation for the observed genetic overlap is the high overlap of symptoms between the two disorders with regard to depressive symptoms. However, BD and MDD still differ largely in course of illness, symptomatology and treatment overall. Another potential explanation is that the association between BD-PRS and MDD case status is due to poor assessment of previous hypomanic symptoms in psychiatric patients. That is, a number of patients classified as having MDD in our sample might be misdiagnosed BD patients with undocumented/undetected hypomanic symptoms. However, a recent study showed that BD-PRS was not associated with hypomania (19). In addition, all subjects in our study were diagnosed by experienced raters with the semi-structured clinical interview SCID-I, which is a valid instrument to detect hypomania symptoms.

Instead, the most likely explanation for the observed association of BD-PRS with increased odds of being diagnosed with MDD is that some of our subjects diagnosed with MDD are actually affected by BD, but have not clinically converted to BD yet [i.e., they are “hidden BD patients” (56)]. A major strength of our study is that we focus on young adults, since numerous prior studies have shown that early age of onset for MDD is a predictor of bipolar conversion (24). This is in line with the fact that the index episode for most BD cases is a depressive episode (22–24). However, the unknown degree to which “hidden BD patients” comprise MDD case samples makes it difficult to distinguish between pleiotropy and truly shared biological pathways in the association of genetic risk for BD with MDD. To further investigate this issue and other open questions such as whether BD-PRS decreases with increasing age in MDD cases, large-scale longitudinal studies of conversion rates for individuals diagnosed with MDD are needed.

### BD-PRS and ADHD

The association between BD-PRS and ADHD case vs. healthy control status is in line with previous findings of high comorbidity and symptom overlap between the two disorders—especially in the age group assessed in the present study (20). However, as with MDD, there are multiple possible explanations for the observed association in the context of previous findings. It is unclear if the high comorbidity of BD and ADHD is simply a result of misdiagnosis due to similarity of symptom complexes, if it is a true comorbidity or whether ADHD is more likely a prodromal manifestation of BD (13). Given that all diagnoses were given based on the results of semi-structured interviews and standardized instruments based on the DSM-IV and DSM-5 and carried out by trained clinicians, we are confident that misdiagnosis in our sample was very unlikely. It is well-documented that a comorbid diagnosis of ADHD is associated with worse outcomes for BD-affected individuals. BD patients with comorbid ADHD have an earlier onset of diagnosed BD, a worse course of illness and a greater burden of other psychiatric comorbid conditions, regardless of whether the ADHD symptoms persist in adulthood or not (14, 57). Duffy (58) proposed that the clinical and biological overlap between BD and ADHD might also be part of a phenotype predicting a specific subtype of BD. In view of the fact that we focused on young adults in our study, our findings might represent a distinct early-onset subtype of BD.

While a recent review only found evidence for a weak association of BD-PRS with ADHD at best (54), various other results support the observed association between BD-PRS and ADHD diagnosis in our study (18–20, 24, 59). However, with regard to the genetic correlation between BD and ADHD, different iterations of the PGC BD-ADHD cross-disorder correlations give different results, and even the correlation between different PGC-BD GWAS phases varies. For example, a larger correlation was observed in Hulzen et al. (20) compared to the later study based on an increased sample by O'Connell et al. (18). In addition, one study has also found evidence for distinct underlying genetic mechanisms (20). Altogether, given the reported genetic association between BD and ADHD, our findings support the assumption that these disorders share genetic underpinnings. Of interest are similar positive genetic correlations of ADHD with early- and late-onset BD. Further research is needed to disentangle the distinct and shared genetic mechanisms of BD and ADHD.

### BD-PRS and ER

Based on the heterogeneity of BD and the unknown composition of risk factors, it is challenging to accurately index individuals with a high propensity to develop BD (23). Most BD patients experience a variety of symptoms, which vary in severity, frequency, and duration and increase until they fulfill full diagnostic criteria (4, 6). However, some risk factors appear to be better indicators than others for propensity to develop BD. The best method to date to quantify risk for BD is the preliminary EPIbipolar (13) used to assign subjects the ER group in this study. Although the risk assessment tool uses key symptom profiles comprising weighted well-documented risk factors associated with later disease manifestation, it is still unclear how well EPIbipolar can measure/predict risk for BD (60). Likewise, EPIbipolar has not yet been tested for an association with BD-PRS. While we could observe a higher average BD-PRS in the ER sample compared to the healthy control group, BD-PRS was not a significant predictor of ER group vs. healthy control status per the corrected significance level. The limited statistical power of this subgroup analysis due to the small sample size of the ER risk group may explain why the weak association did not reach the level of statistical significance. These results underline the need for further research with larger sample sizes, envisaged by BipoLife (40, 41). The dichotomized EPIbipolar threshold for elevated risk used to assign subjects to the ER group (EPIbipolar risk, high-risk and ultra-high-risk group vs. no risk group classification) may also play a role in the negative findings. Exploratory analyses revealed a higher association between the BD-PRS and ER group vs. healthy control status when only subjects who fell into the EPIbipolar high-risk and ultra-high-risk groups were included. Therefore, the lack of association between BD-PRS and ER group status might be a result of an underestimated threshold that leads to an information bias (2, 9). A more stringent threshold for EPIbipolar results and larger sample sizes may enable the detection of an underlying association of BD-PRS with ER BD risk status.

### Limitations

A limitation of the present study is the number of participants assessed, particularly when analyzing the three risk groups separately. While our power analysis indicated an adequate power to detect effects as described in the literature for BD case–control samples (31) for the combined risk group, power was limited for analyses of the individual subgroups. In order to detect more subtle effects or investigate the characteristics of subgroups in more detail, larger samples are needed. Another limitation is the predictive power of BD-PRS. While BD-PRS were derived from the largest GWAS of BD to date, substantially larger discovery samples are needed to fully leverage the predictive power of PRS. Additionally, PRS capture only common genetic variations and their effects on risk—rare variants may also play a role in BD risk. We also acknowledge that, by using the best-fit approach implemented in PRSice, the observed variance explained by PRS (pseudo *R*^2^; PRS.*R*^2^.adj = 0.0174038) is likely an overestimation of the true value. Finally, follow-up studies are required to determine how many high-risk participants convert to BD to determine the predictive validity of the BD-PRS associations.

## Conclusion

In conclusion, we found associations between increased genetic risk for BD and increased odds of MDD and ADHD in young adulthood, but not for odds of ER group status. While PRS only explain a relatively small proportion of the variance of BD, the results of our study indicate that BD-PRS may be still useful for early identification and risk stratification in the future. Currently, the predictive power of psychiatric PRS is still too limited for clinical application (61). However, future, exponentially larger GWAS will substantially increase the signal reliably captured and increase the predictive power of PRS (39). Furthermore, methodological advances of risk scoring methods [e.g., by improved algorithms or inclusion of rare variants, will further improve genetic risk prediction (62)]. Given the comorbidity of MDD and BD, lack of early diagnosis, and the fact that a first onset MDD diagnosis may actually represent an early-onset BD phenotype, further work in longitudinal studies could explore how many high-risk individuals convert to BD. In this regard, it would be interesting to see if those who convert to BD are also those who have a high BD-PRS score. Additionally, a stricter definition of ER status to best reflect conversion risk could contribute to improved BD risk prediction.

## Data Availability Statement

The datasets presented in this article are not readily available because participants of the study did not give permission to publish their genome-wide data, based on obvious conflict with General Data Protection Regulation (OJ L 119, 04.05.2016; cor. OJ L 127, 23.5.2018.; https://gdpr-info.eu/). Requests to access the datasets should be directed to Andreas Reif, Andreas.Reif@kgu.de.

## Ethics Statement

The studies involving human participants were reviewed and approved by Ethics Committee of the University Hospital Frankfurt am Main, Germany. The patients/participants provided their written informed consent to participate in this study.

## Author Contributions

SB, KP, SM, OG, MBr, and NB-K: acquisition of data. AR, SK-S, AP, SM, TK, OG, VO, MBa, and TS: critical revision. SB, TK, SK-S, AR, SM, and AP: drafting of manuscript. SB and TK: analysis and interpretation of data. All authors contributed to the article and approved the submitted version.

## Conflict of Interest

The authors declare that the research was conducted in the absence of any commercial or financial relationships that could be construed as a potential conflict of interest.
